# No evidence that middleborns feel less close to family and closer to friends than other birth orders

**DOI:** 10.1016/j.heliyon.2020.e03825

**Published:** 2020-05-07

**Authors:** Veronika Simanko, Ben Rimmer, Thomas V. Pollet

**Affiliations:** a Dept. of Psychology, Northumbria University, Newcastle upon Tyne, UK; b Dept. of Evolutionary Anthropology, University of Vienna, Austria; c Newcastle University, Newcastle Upon Tyne, UK

**Keywords:** Psychology, Family, Friendship, Born to Rebel, Birth order

## Abstract

Middleborns have been argued to be the neglected birth order. The present study aimed to test whether the emotional closeness to parents, siblings or friends differed between middleborns and otherborns, across two different datasets. Using a between family design this study accounted for gender, nationality, educational attainment, age and sibship size via matching. We found no evidence to suggest that middleborns differ from otherborns in familial sentiment. The sign of closeness to friends was in the opposite direction than predicted, with middleborns being less close than other birth orders. The findings are discussed with reference to current literature on birth order and familial sentiment.

## 1 Introduction

Birth order effects have been argued to be important for individual differences, throughout the history of psychology (Adler, 1954; Galton, 1874). In the 1990s, Sulloway rekindled the interest in birth order effects (Sulloway, 1995, 1996), after previous suggestions that studying birth order effects amounted to a waste of time (Ernst and Angst, 1983). Sulloway's model is based on the finding that children in the same family tend to develop very different personalities (Sulloway, 1996, 2010). This is presumably due to the experience of various non-shared environmental factors (McGue and Bouchard Jr., 1998; Plomin and Daniels, 1987), such as birth order (Sulloway, 1996, 2010). Using an evolutionary theoretical model of parental investment (Trivers, 1974), Sulloway argued that niche picking strategies in development should closely align with birth order (Sulloway, 1995, 1996). Firstborns have been argued to be most achieving and most conscientious, according to Sulloway's model of “niche picking”. For firstborns, the best strategy to maximize parental investment is to conform. On the other hand, laterborns confronted with less remaining niches to pick, should be most “rebellious” in order to stand out (Paulhus et al., 1999; Sulloway, 1996).

However, it has been suggested that we need to further refine the classification of “laterborns” into “middleborns” and “lastborns”, even though many earlier studies just differentiate between firstborns and laterborns (Saroglou and Fiasse, 2003). Yet, middleborns might be quite different from other birth orders. This argument is based on a lack of uniqueness and low parental investment attributed to the birth position of the middleborn, leading them to be coined as the “neglected birth order” (Kidwell, 1982; Salmon and Daly, 1998; Salmon et al., 2012). Firstborns have an advantage as, for a given period, they do not have to compete against another sibling for parental investment (Salmon, 2015; Salmon and Daly, 1998; Salmon et al., 2012). In addition, from an evolutionary perspective, it has been argued that parents could benefit from prioritising investment in the oldest child (see Lewis and Kreitzberg, 1979; Draper and Hames, 2000; Salmon and Daly, 1998; Sulloway, 1996). For example, firstborns, when they survive a period of heightened mortality during childhood, will have higher reproductive potential than other birth orders (Trivers, 1974). The birth position of the lastborn child has also been argued to elicit heightened investment by parents, since this child typically has the highest need (Daly and Wilson, 1984; Kidwell, 1982; Rohde et al., 2003; Salmon and Daly, 1998). Therefore, parental investment has been argued to follow a curvilinear pattern (Hertwig et al., 2002) with middleborns receiving relatively less investment than firstborns and lastborns, given that middleborns only have a limited period where they are the only child for parents to invest in.

In support of the suggested lack of preferential treatment of middleborns, Kidwell (1982) found middleborns to have significantly lower self-esteem than otherborns. Further support comes from Suitor and Pillemer (2007)'s finding that middleborns are underrepresented in mothers' choices regarding closeness to their children. In fact, middle-borns were 80% less likely than a lastborn to be chosen as the child the mother feels closest to. Lindert (1977) found middleborns to receive about 10% less care in terms of total child care hours than their first- or lastborn siblings. While firstborns are the only child in the family for a certain period of time before the second child is born, lastborns are likely to remain the only child in the household after older siblings leave home. In contrast, middleborns, most likely, must share parental resources at all times during their development (Hertwig et al., 2002; Lindert, 1977; Price, 2008). In sum, middleborns have been argued to end up with fewer (parental) resources and quality time than other birth orders (Kidwell, 1982; Rohde et al., 2003; Salmon and Daly, 1998; Sulloway, 1996).

The effects of this lowered parental investment have been argued to be reflected in later adult life (e.g., Kidwell, 1982; Rugala and Nystul, 1998; Salmon, 1999; Salmon and Daly, 1998; Sulloway, 2010). Several studies demonstrated that, all else being equal, middleborns showed lower closeness to their family, especially their parents, than other birth orders (e.g., Kennedy, 1989; Kidwell, 1981, 1982; Salmon, 2003; Salmon and Daly, 1998; Ziv and Hermel, 2011). For example, Salmon and Daly (1998) found middleborns, compared to other birth positions, to be more likely to turn to a sibling, than a parent for support. In addition, middleborns were the least likely birth order to name their mother as the person they feel closest to (also see Rohde et al., 2003; Salmon, 2003; Salmon et al., 2016). Consequently, given middleborns' proposed lower familial sentiment, they were also suggested to be relatively more invested in friendships than family, compared to otherborns (Mysterud et al., 2006; Salmon, 2003; Salmon et al., 2016). In line with this prediction, Salmon (2003) found middleborns to express a more positive attitude towards their friends than other birth orders (also see Salmon et al., 2016). Further support comes from a study by Mysterud et al. (2006), who found that middleborns spend more on gifts for their friends than other birth orders.

However, in an attempt to replicate Salmon and Daly's (1998) findings, Hardman et al. (2007) did not find any evidence for the proposed middleborn effect in familial sentiment in children or adults. This is supplemented by several other studies finding no middleborn effects in familial sentiment (Euler and Michalski, 2007; Pollet and Nettle, 2007), or only finding support by using specific study designs, such as within-family designs but not between-family designs (Pollet and Nettle, 2009), or a neglected middleborn effect only being found in conjunction with another variable, e.g. mother's age (Rohde et al., 2003). More generally, birth order effects, such as Sulloway's (1996) findings on the effects of birth order on social attitudes and other aspects of personality, have not been corroborated by several studies (e.g., Beer and Horn, 2000; Bleske-Rechek and Kelley, 2014; Dunkel et al., 2009; Freese et al., 1999; Førland et al., 2012; Marini and Kurtz, 2011; Rohrer et al., 2015). Altogether, such findings suggest these birth order effects might prove to be elusive and might only show in very specific circumstances or research designs (Freese et al., 1999; Marini and Kurtz, 2011; Rohde et al., 2003).

Given the conflicting findings, it is important to further investigate the proposed effects of birth order, and test the degree to which previous findings on neglected middleborns, i.e. birth order effects on familial sentiment, are upheld. Thus, the present study aims to test whether adult middleborns rate parents, siblings or friends as lower in emotional closeness than otherborns, across two datasets. Using a between family design, covariates such as gender, age, sibship size, nationality and educational attainment were accounted for via matching middleborns to otherborns on these variables. Alternatively, the null hypothesis is that there are no notable differences between middleborns and otherborns in ratings of emotional closeness to parents, siblings or friends.

## 2 Study 1

### 2.1 Method

#### 2.1.1 Participants

In order to obtain a large sample which also included non-students, participants were recruited via the personal networks of students enrolled at a large Dutch university. Students received credits in exchange for returning completed questionnaires. More details can be found (Pollet et al., 2013; Pollet et al., 2018). There are a large number of German participants as this study proved popular with German-speaking students who had fewer options of studies to participate in exchange for credit. 458 surveys were processed (301 women, M=30.97 years, SD=14.55 years, 3 participants did not report age or gender). Due to non-response and the criterion of having a biological sibling, the working sample consisted of 297 individuals.

#### 2.1.2 Procedure and measures

After providing informed consent, participants completed a paper-based survey in either German or Dutch depending on the participant's language preference. Participants first provided some basic sociodemographic data, including age, gender, nationality and educational attainment. They also indicated their birth order (*What is your position in the birth order?*: firstborn, middleborn, lastborn - translated to Dutch/German). They then completed a questionnaire on their social networks. They were first asked to list living relatives, after which they were asked to list friends and acquaintances. They were instructed to go through any of their address lists and list all of the people for whom they had contact details. Participants were instructed to list any contact they considered to be a personal relationship. For each individual they indicated the type of network member (e.g., the family relationship they had to them) and rated the emotional closeness to that social network member (*On a scale of 1-10 (where 10 is very close) please say how close the person is to you in terms of how you feel about them* – note that some participants deviated and also used 0.). Emotional closeness has been previously argued to measure tie strength (e.g., Hill and Dunbar, 2003; Marsden and Campbell, 1984; Roberts et al., 2009; Sutcliffe et al., 2012). The survey also included some other measures not used and discussed here (for example, a questionnaire on loneliness, more details in Pollet et al., 2018). The procedure was approved by the local ethics committee at the University of Groningen.

#### 2.1.3 Analyses

We used R (R Development Core Team, 2008). First, we performed matching of middleborns to otherborns via genetic matching (Diamond and Sekhon, 2013). Genetic matching solves the finding of matches on covariates via a genetic search algorithm. Individuals were matched on the following covariates: gender, nationality, educational attainment, age, and number of siblings. This approach of matching allows us to reduce the effects of confounding in our observational data (e.g., Austin, 2011) and creates a powerful test for the hypothesis. Via this way of matching, we were able to match *all* the middleborns (N=59) to otherborns (N=59). For this matched sample, we perform ordinary least squares regressions. We also report Bayes Factors (Kass and Raftery, 1995), which allow to weigh the evidence for the alternative versus the null hypothesis. Our analysis document and code, including further analyses, can be found at https://osf.io/6jpu5/.

### 2.2 Results

**Figure 1:**
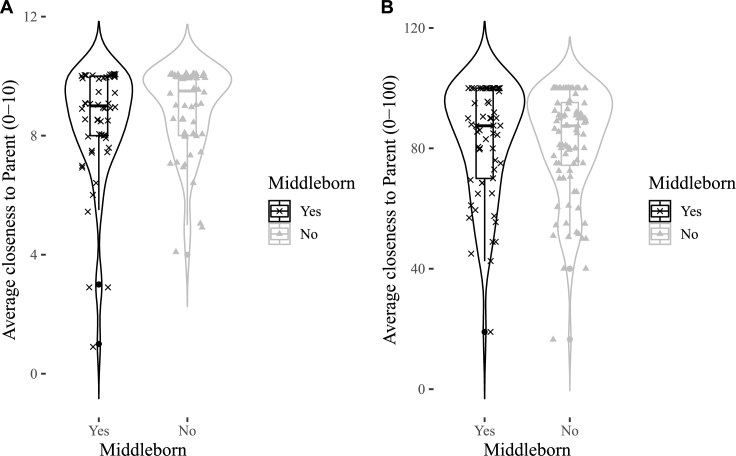
Violin plots for middleborns and otherborns on emotional closeness to a parent. A = Study 1, B = Study 2.

**Figure 2:**
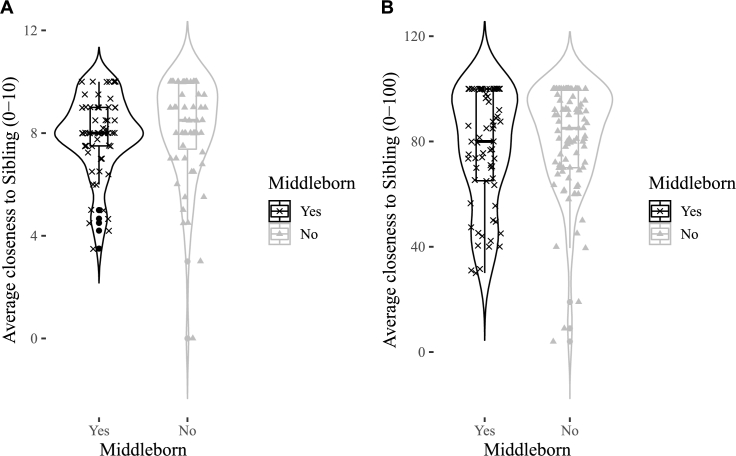
Violin plots for middleborns and otherborns on emotional closeness to a sibling. A = Study 1, B = Study 2.

**Figure 3:**
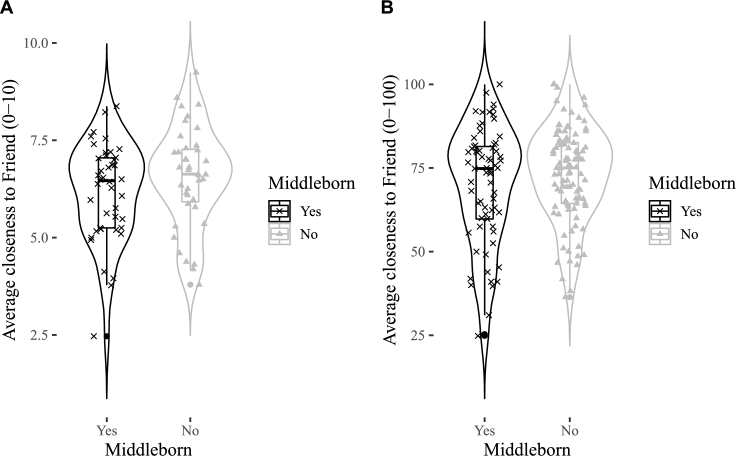
Violin plots for middleborns and otherborns on emotional closeness to a friend. A = Study 1, B = Study 2.

Figs. 1A, 2A, 3A show the distributions of emotional closeness to a parent, sibling, friend by birth order. There were consistently no statistically significant effects of being a middleborn on average emotional closeness to a parent, sibling, or friend (Table 1, all p's>.6). The sign of closeness to friends is in the opposite direction than predicted.

**Table 1:** OLS regressions for Study 1.

	Parent closeness	Sibling closeness	Friend closeness
	(1)	(2)	(3)
Middleborn	−0.092	−0.126	−0.160
Constant	8.494^⁎⁎⁎^	8.041^⁎⁎⁎^	6.329^⁎⁎⁎^
*N*	109	118	81
R^2^	0.001	0.001	0.004
Adjusted R^2^	−0.009	−0.007	−0.008
Residual Std. Error	1.830 (df = 107)	1.647 (df = 116)	1.202 (df = 79)
F Statistic	0.067 (df = 1; 107)	0.173 (df = 1; 116)	0.337 (df = 1; 79)

^⁎^p <.05; ^⁎⁎^p <.01; ^⁎⁎⁎^p <.001.

Bayes Factors favour the null hypotheses, i.e. no difference, for emotional closeness to parents, siblings or friends with varying support (Bayes factors 1.91, 4.03, and 1.86).

## 3 Study 2

### 3.1 Method

#### 3.1.1 Participants

525 participants (63.4% women, M=27, SD=10.09, range 18 to 83 years) took part in an online survey on social networks in English or Dutch. The sample is described in more detail in (Molho et al., 2016). From this dataset, we selected participants who reported having at least one biological sibling (N=455).

#### 3.1.2 Procedures and measures

Participants were first asked to list all people with whom losing contact forever would be upsetting (*“We would like you to think of the people who are most important to you, and to imagine not being able to speak or to see these people ever again”*). After completing measures of support for these members (not discussed here, see Molho et al., 2016), participants reported how emotionally close they felt to each network member on a 0 to 100 scale. Emotional closeness is considered the most reliable indicator of tie strength (Marsden and Campbell, 1984). We calculated the average emotional closeness to (biological) parents, (biological) siblings and friends. Participants indicated their birth order (*This question is about your birth order. Are you a …?*: Firstborn (or only child), Middleborn, Lastborn). Note that there were other measures not used in our analyses and therefore discussed here (for example, a questionnaire on personality, more details in Molho et al., 2016). The procedure was approved by the local ethics committee at the VU University Amsterdam.

#### 3.1.3 Analyses

The analytical procedure is the same as in Study 1, we were able to match all middleborns (N=74) to otherborns (N=109) on education, native language, number of siblings, age and gender. Note that now we do not have a 1:1 to match, weights are thus applied in our regression analyses.

### 3.2 Results

There was no suggestion that respondents' birth order influenced ratings of closeness to parents or siblings (both *p*'s >.2, Table 2). However, there was a weak statistical trend for a birth order effect in reported closeness to friends (F(1,166)=3.01, p=.085). Yet, this effect is in the *opposite* direction as predicted.

**Table 2:** OLS regressions for Study 2.

	Parent closeness	Sibling closeness	Friend closeness
	(1)	(2)	(3)
Middleborn	−4.294	−3.986	0.075
Constant	73.905^⁎⁎⁎^	81.003^⁎⁎⁎^	82.665^⁎⁎⁎^
*N*	168	163	153
R^2^	0.018	0.009	0.00000
Adjusted R^2^	0.012	0.003	−0.007
Residual Std. Error	15.620 (df = 166)	20.208 (df = 161)	16.507 (df = 151)
F Statistic	3.005 (df = 1; 166)	1.530 (df = 1; 161)	0.001 (df = 1; 151)

^⁎^p <.05; ^⁎⁎^p <.01; ^⁎⁎⁎^p <.001.

Bayes factors again pointed to the null hypothesis rather than the alternative hypothesis (Parent: 5.49, Sibling: 2.41, Friend: 2.32).

## 4 Discussion

Our findings suggested no measurable effect of being a middleborn on average emotional closeness to family. Interestingly, with average emotional closeness to friends, if anything the effect would be in the opposite direction to Salmon and Daly (1998, 2003), and Rohde et al. (2003), who found middleborns to be closer to their friends compared to other birth orders.

Since our study has a matched design, allowing us to rule out potential confounds such as the number of siblings, age, gender and educational attainment, our results should be unaffected by these previously known factors (Rohde et al., 2003; Salmon and Daly, 1998). With a view to the ongoing debate regarding within- vs. between-family design (Michalski and Shackelford, 2001; Rodgers et al., 2000), we believe that both designs may suffer from confounds (see Steelman and Powell, 1985; Michalski and Shackelford, 2001; Paulhus et al., 1999; Pollet and Nettle, 2009). Further research is necessary to fully uncover the choice of design's influence on the findings (Pollet and Nettle, 2009). However, Steelman and Mercy (1980) and Steelman and Powell (1985) argued that birth order effects should be detectable by between-family designs, if they were to have an important effect more broadly on behavior in society. Furthermore, even using a within-family design, studies have failed to replicate other birth order findings (see Freese et al., 1999; Bleske-Rechek and Kelley, 2014; Rohrer et al., 2015). Therefore, we believe, given the robustness of our design against confounds, a difference in closeness to family in adulthood between middleborns and otherborns should have been detectable if present. For now, we conclude that our study adds to the literature not supporting birth order effects - in our case we found no evidence for a “neglected middleborn” effect.

A potential explanation for why other, previous studies have found a “neglected middleborn effect” could be the age composition of the sample enhancing the middleborn effect (Pollet and Nettle, 2009). While students often still compete with their siblings for their parents' resources, this kind of sibling competition declines in adulthood (Pollet and Nettle, 2009). As many studies used data from undergraduate populations (Kidwell, 1982; Rohde et al., 2003; Salmon, 2003; Salmon and Daly, 1998), they might have captured a middleborn effect that only occurs in this particular family stage where adolescent siblings are competing for their parents' resources (Pollet and Nettle, 2009). Therefore, the middleborn effect in an adult sample might be negligible and therefore not detectable using a between-family design (Pollet and Nettle, 2009) or its purported effect could be much smaller. Future research might benefit from adopting a life course perspective and examining whether the “neglected middleborn” effect is limited to certain life stages. In this context, Hardman et al. (2007)'s demonstrated the absence of a “middleborn effect” in both a sample of children and adults. However, we are lacking studies examining these effects throughout the life span.

Alternative explanations could also account for why we did not find support for a neglected middleborn effect. Rohde et al. (2003) found that a mother's age was of importance for the middleborn effect. Only middleborns with mothers that were older than 27 years old at the time of their birth, were found to be least likely to name their mother as person they feel closest to. One reason for this might be the notion that older mothers tend to focus their investment on the youngest children, due to their low residual reproductive potential - a phenomenon known as terminal investment (e.g., Williams, 1966; Part et al., 1992). Age spacing has also been shown to influence middleborn effects, with a spacing of two years to the adjacent siblings showing the strongest effects (Kidwell, 1981, 1982). This age spacing might be especially unfavorable when distributing parental investment during development, thereby diminishing familial sentiment in middleborns (see Kidwell, 1981, 1982; Sulloway, 2010). If there is narrow sibling spacing, then it is possible that there would only be a negligible effect on familial sentiment (see Lindert, 1977; Kidwell, 1981). On the other hand, a wider spacing between siblings might facilitate caregiving, as older children become more autonomous and might actually help out in the household and with childcare (see Kidwell, 1981; Steelman et al., 2002). As a consequence, a wider spacing might lead to obscure any effects (see Kidwell, 1981). Perhaps more important than age spacing effects is family constellation. It is unclear to which degree biological relatedness is relevant for birth order effects in family relationships. While some studies have suggested that the sibling relationships differ between fully related and not fully related siblings (e.g., White and Riedmann, 1992; Pollet, 2007), it is unclear what the impact will be of changing family dynamics on birth order effects. Given that family constellations have been dramatically changing (e.g., Bumpass and Lu, 2000), it is unclear how this will affect birth order effects on familial sentiment in the future. It is possible that more recent findings not supporting a neglected middleborn effect are already reflecting broader changes in how family structures are rapidly changing in society.

It is important to note that there are multiple limitations to our studies. First, it must be recognised that our samples are limited to a Western context (e.g., Arnett, 2008; Henrich et al., 2010; Pollet and Saxton, 2019). While we made an effort to move beyond a typical student sample (Gallander Wintre et al., 2001), our sample remains much younger than the general population. Second, while we were able to match siblings on many relevant traits (e.g., sibship size), there could be other, unmeasured, confounds which obscure the presence of a birth order effect. For example, as discussed above, birth spacing could be a factor of importance and we did not capture this variable. That being said, birth spacing should attenuate any baseline effect and studies have reported middleborn effects without accounting for age spacing (e.g., Saroglou and Fiasse, 2003). Third, in both cases we relied on a single item measure to establish emotional closeness, which we averaged across categories. Given that sibling relationships are multifaceted (Cicirelli, 1991), future research would benefit from using measures which capture the dimensions of a sibling relationship more comprehensively. Finally, our study did not collect in depth measures on all family members or use a round-robin design whereby all family members rate each other.

To summarise, in addition to the discussed evidence, the null findings of the present study support the suggestion that the “neglected middleborn” effect in social relationships might not be robust (e.g., Hardman et al., 2007; Pollet and Nettle, 2007). This is in line with other studies finding no measurable effect of birth order on personality (e.g., Ernst and Angst, 1983; Rohrer et al., 2015), risk-taking (Lejarraga et al., 2019) and socio-political attitudes (e.g., Freese et al., 1999; Førland et al., 2012). Thus, we argue that at present there is no strong evidence that in adulthood, middleborns feel less close to their family and closer to friends than other birth orders. Future research may benefit from turning away from birth order in favour of other variables (Ernst and Angst, 1983), such as educational attainment, gender, social class, and family size to explain purported birth order effects (Bleske-Rechek and Kelley, 2014; Ernst and Angst, 1983; Hardman et al., 2007; Steelman et al., 2002).

## Declarations

### Author contribution statement

V. Simanko, B. Rimmer: Conceived and designed the experiments; Wrote the paper.

T.V. Pollet: Conceived and designed the experiments; Performed the experiments; Analyzed and interpreted the data; Contributed reagents, materials, analysis tools or data; Wrote the paper.

### Funding statement

This research did not receive any specific grant from funding agencies in the public, commercial, or not-for-profit sectors.

### Competing interest statement

The authors declare no conflict of interest.

### Additional information

Data associated with this study has been deposited at The Centre of Open Science, OSF under https://osf.io/6jpu5/.
